# Sulfur reduces the root-to-shoot translocation of arsenic and cadmium by regulating their vacuolar sequestration in wheat (*Triticum aestivum* L.)

**DOI:** 10.3389/fpls.2022.1032681

**Published:** 2022-10-05

**Authors:** Gaoling Shi, Huan Liu, Dongmei Zhou, Huimin Zhou, Guangping Fan, Wei Chen, Jiangye Li, Laiqing Lou, Yan Gao

**Affiliations:** ^1^ Key Laboratory of Agro-Environment in Downstream of Yangtze River Plain, Ministry of Agriculture and Rural Affairs of the People’s Republic of China, Institute of Agricultural Resources and Environment, Jiangsu Academy of Agricultural Sciences, Nanjing, China; ^2^ School of the Environment and Safety Engineering, Jiangsu University, Zhenjiang, China; ^3^ Luhe Agro-Environment Experimental Station of National Agricultural Observation and Research Station, Nanjing, China; ^4^ College of Life Sciences, Nanjing Agricultural University, Nanjing, China; ^5^ State Key Laboratory of Pollution Control and Resource Reuse, School of the Environment, Nanjing University, Nanjing, China

**Keywords:** cadmium, wheat, arsenic, subcellular distribution, phytochelatin, TaABCC1

## Abstract

Accumulation of arsenic (As) and cadmium (Cd) in wheat grain is a serious threat to human health. Sulfur (S) can simultaneously decrease wheat grain As and Cd concentrations by decreasing their translocation in wheat; however, the mechanisms are unclear. We conducted hydroponic experiments to explore the mechanisms by which S modulates As and Cd translocation and their toxicity in wheat. Wheat seedlings were grown in deficient sulfate (2.5 µM) or sufficient sulfate (1.0 mM) nutrient solutions for 6 days and then exposed to zero (control), low As+Cd (1 µM As plus 0.5 µM Cd), or high As+Cd (50 µM As plus 30 µM Cd) for another 6 days. Compared with the control, plant growth was not affected by low As+Cd, but was significantly inhibited by high As+Cd. In the low As+Cd treatment, S supply had no significant effect on plant growth or root-to-shoot As and Cd translocation. In the high As+Cd treatment, sufficient S supply significantly alleviated As and Cd toxicity and their translocation by increasing phytochelatin (PC) synthesis and the subsequent vacuolar sequestration of As and Cd in roots, compared with deficient S supply. The use of _L_-buthionine sulfoximine (a specific inhibitor of γ-glutamylcysteine synthetase) confirmed that the alleviation of As and Cd translocation and toxicity in wheat by S is mediated by increased PC production. Also, *TaHMA3* gene expression in wheat root was not affected by the As+Cd and S treatments, but the expression of *TaABCC1* was upregulated by the high As+Cd treatment and further increased by sufficient S supply and high As+Cd treatment. These results indicate that S-induced As and Cd subcellular changes affect As and Cd translocation mainly by regulating thiol metabolism and *ABCC1* expression in wheat under As and Cd stress.

## Introduction

Heavy metal/metalloid pollution in soil-crop systems is a potential threat to human health. Arsenic (As) and cadmium (Cd) are ubiquitously distributed, highly toxic, and readily transferred from contaminated soil to the food chain (Zhao and Wang, 2020). Long-term exposure to these two toxic elements can cause lung disease, skin disease, kidney disease, and Itai-Itai disease (Godt et al., 2006; Balali-Mood et al., 2021). Wheat (*Triticum aestivum* L.) is one of the most important staple foods and ranks first in terms of global consumption, accounting for about 28% of cereal consumption annually (Food and Agriculture Organization, 2021). Compared with other grain crops such as barley and maize, wheat grain has greater ability to accumulate Cd (Yang et al., 2014; Rizwan et al., 2016). In alkaline soil, wheat can still accumulate considerable amounts of Cd in its grain at concentrations even higher than those seen in rice (Yang et al., 2020). As a result, wheat and wheat-based products are major dietary sources of Cd (Greger and Löfstedt, 2004; Punshon and Jackson, 2018). Although wheat grain has lesser As accumulation than rice (Williams et al., 2007; Bhattacharya et al., 2010; Saeed et al., 2021), high As concentration was commonly detected in the grain of wheat grown in As-contaminated soils (Duncan et al., 2017; Suman et al., 2020). Arsenic is typically present in wheat grain in an inorganic form (Zhao et al., 2010; Shi et al., 2013; Rasheed et al., 2018), which is more toxic to humans than the organic form (Styblo et al., 2000; Sun et al., 2008). Risks to human health as a result of inorganic As exposure through consumption of wheat have been reported in India (Suman et al., 2020), Pakistan (Rasheed et al., 2018), and Argentina (Sigrist et al., 2016). Therefore, there is a need for strategies to decrease As and Cd accumulation in the grain of wheat grown in As and Cd co-contaminated soils.

Most previous studies focused on the uptake and translocation of As and Cd individually in soil-crop systems, and many mitigation strategies have been employed to reduce As or Cd accumulation in food crops. However, farmland contaminated with both As and Cd has been reported worldwide (Wan et al., 2020; El-Naggar et al., 2021). Simultaneous decreases in As and Cd accumulation in crops are a challenge because of their opposite behaviors in soil (Honma et al., 2016; Qiao et al., 2018). For example, liming of acidic soils with lime or biochar is effective in decreasing Cd accumulation in crops, but not for As (Zhu et al., 2016; Chen et al., 2018). Water and phosphorous fertilizer management exert opposite effects on As and Cd bioavailability to plants (Lee et al., 2016; Wan et al., 2019; Khan et al., 2020). Likewise, the effect of sulfate on As and Cd bioavailability in wheat rhizosphere soil is inconsistent; interestingly, sulfur (S) supply decreased As and Cd accumulation in wheat grain by decreasing their translocation in wheat plants grown in As and Cd co-contaminated soil (Shi et al., 2020). However, the mechanism underlying S-mediated regulation of As and Cd translocation in wheat is largely unknown.

The subcellular distribution of As and Cd significantly affects the concentration of free As and Cd ions in plant cells, modulating As and Cd translocation and toxicity to plants (Srivastava et al., 2016; Cao et al., 2018; Huang et al., 2021). In rice, S supply alters the Cd subcellular distribution by regulating *OsHMA3* (OsHMA3, a transporter for Cd sequestration into root vacuoles) expression (Cao et al., 2018). However, the TaHMA3 transporter may not be able to transport Cd in wheat (Zhang et al., 2020). Therefore, whether S can alter Cd subcellular distribution in wheat by another pathway warrants further investigation. The ATP-binding cassette transporters ABCC1 and ABCC2 are reportedly involved in the vacuolar sequestration of Cd in *Arabidopsis thaliana* (Park et al., 2012). In contrast to HMA3, which transports the ionic form of Cd, the ABCC transporters transport Cd-phytochelatin (PC) conjugates (Zhang et al., 2018). PCs also have a high affinity for As binding to their thiol groups. AtABCC1 and AtABCC2 transport both Cd-PC and As-PC complexes (Park et al., 2012; Song et al., 2014a). Therefore, the magnitudes of PC synthesis and *ABCC* expression in plants may determine As and Cd mobility in roots. In a recent study, 18 wheat ABCC proteins were identified; a phylogenetic analysis showed that only TaABCC1 is an ortholog of AtABCC1 and AtABCC2 (Bhati et al., 2015). However, the relationship between *TaABCC1* expression and As and Cd translocation in wheat is unclear.

Sulfur is a vital macronutrient for plant growth and responses to abiotic and biotic stresses (Tao et al., 2018). Numerous crucial S-containing compounds are derived directly or indirectly from this element, including cysteine (Cys), glutathione (GSH), PCs, and nicotinamide (Na and Salt, 2011). We hypothesized that S supply alters the subcellular distribution of As and Cd by upregulating PC synthesis and *TaABCC1* expression, thus reducing the root-to-shoot translocation of As and Cd in wheat plants. Accordingly, in this study, we exposed wheat seedlings to both As and Cd under various levels of S supply in solution culture to investigate the influence of S on As and Cd uptake, translocation, and subcellular distribution, as well as plant growth. Furthermore, the effect of S supply on GSH and PC synthesis, *TaABCC1* and *TaHMA3* expression, and their relationships with As and Cd translocation in wheat seedlings were analyzed. The findings provide theoretical guidance for bioremediation of As and Cd in wheat plants to reduce environmental and health hazards.

## Materials and methods

### Plant culture and treatment

Wheat (*Triticum aestivum* L. cv. Jinmai 85) seeds were surface-sterilized in 10% (w/w) hydrogen peroxide for 10 min, washed thoroughly with clean water and soaked in distilled water for 12 h, and germinated in wet quartz sand for 3 days. After germination, uniform seedlings were selected and transplanted into a black plastic beaker (twelve seedlings per beaker) with 1.1 L of 0.5 mmol L^–1^ CaCl_2_ solution for 3 days, and then the seedlings with residual endosperm removed were transferred into nutrient solution with deficient sulfur (S1: 2.5 µM sulfate, the concentration of sulfate in micronutrients) or sufficient sulfur (S2: 1.0 mM sulfate, the concentration of sulfate in full nutrient solution). The sufficient S nutrient solution (full nutrient solution) contained 1.0 mM Ca(NO_3_)_2_, 1.5 mM KCl, 1 mM MgSO_4_, 0.2 mM KH_2_PO_4_, 1.5 mM CaCl_2_, 1 µM H_3_BO_3_, 0.05 µM (NH_4_)_6_Mo_7_O_24_, 1 µM ZnSO4, 1 µM MnSO4, 0.5 µM CuSO_4_, and 40 µM FeNa_2_-EDTA (Ren et al., 2012). For deficient S supply, MgCl_2_ was instead of MgSO_4_. Nutrient solution was refreshed every 3 days, and solution pH was adjusted to 6.0 using HCl or NaOH. Beakers were placed in a growth cabinet with a 12/12-h light/dark photoperiod and 25/20°C day/night temperatures.

After growing the wheat seedlings in deficient- or sufficient-S nutrient solution for 6 days, they were exposed to zero (0 µM As plus 0 µM Cd; control), low (1 µM As plus 0.5 µM Cd; low As+Cd), or high As and Cd (50 µM As plus 30 µM Cd; high As+Cd) for another 6 days maintained in deficient- or sufficient-S nutrient solution under the above-mentioned growth conditions. The concentrations of As and Cd selected were based on the results of preliminary toxicity assays. Arsenic at 1–10 µM or Cd at 0.5–5 µM has no visual negative effect on the growth of wheat seedlings after 6 days; and As at 50 µM or Cd at 30 µM both caused about 40% inhibition in root elongation (**Figure S1**). Furthermore, to substantiate the inhibition by S of As and Cd translocation in wheat plants by increasing GSH and PC production under As and Cd stress, a GSH synthetase inhibitor, _L_-buthionine sulfoximine (BSO, 0.25 mM) was added to high As+Cd + deficient S supply and high As+Cd + sufficient S supply. The concentration of BSO used in this study was set according to previous studies (Tang et al., 2016; Shi et al., 2017). The effects of the two treatments were compared with that of high As+Cd + deficient S-supply plants. The treatments were arranged in a completely randomized block design with three replicates and twelve plants per beaker. The sources of As and Cd were Na_2_HAsO_4_ and CdCl_2_, respectively.

### Determination of plant growth and concentrations of As and Cd

The lengths of roots (the average value of the two longest roots of each plant) and shoots were measured using a ruler before and after 6 days of As+Cd treatment. After measurement of root and shoot length, wheat plants were harvested. The roots were rinsed in deionized water and immersed in an ice-cold desorption solution containing 1 mM K_2_HPO_4_, 0.5 mM Ca(NO_3_)_2_, and 5 mM MES (pH 6.0) for 10 min to remove root surface-adsorbed As and Cd (Xu et al., 2022). Roots and shoots were separated, washed with distilled water, blotted dry, and weighed. Root and shoot samples were divided into two sets. One set for biochemical analysis was immediately frozen in liquid nitrogen and stored at –80°C, and the other set was dried to a constant weight at 60°C for total As and Cd analysis. The dried samples were weighed and digested with HNO_3_–H_2_O_2_ (2:1, v/v) at 125°C (Shi et al., 2020). Arsenic and Cd concentrations were determined by inductively coupled plasma mass spectroscopy (ICP-MS, Perkin Elmer NexION 2000, Waltham, MA). For quality control, reagent blanks and a certified reference plant material (Orange Leaves GBW10020, National Research Center for Standards, China) were included in the analysis. The recovery rates of the standard reference materials for As and Cd were 92–98% and 95–101%, respectively.

### Determination of As and Cd subcellular distribution

Frozen root and shoot tissues were powdered in liquid nitrogen and extracted with 10 mL of extraction solution containing 50 mM Tris-HCl buffer (pH 7.5), 0.25 mM sucrose, and 1.0 mM DL-dithioerythritol. The homogenate was centrifuged at 1500 *× g* for 15 min at 4°C; the residue was considered the cell-wall fraction. The supernatant was centrifuged at 20,000 *× g* for 30 min at 4°C (Su et al., 2014; Xiao et al., 2020). The supernatant and the residue were referred to as the soluble and organelle fractions, respectively. These fractions were dried to constant weight at 60°C and digested with HNO_3_–H_2_O_2_. Arsenic and Cd concentrations were determined by ICP-MS (Shi et al., 2020).

### Analysis of non-protein thiol compounds

Non-protein thiol compounds in wheat samples were extracted and analyzed as described previously (Wu et al., 2013; Shi et al., 2017). In short, frozen plant samples were ground into powder in liquid nitrogen and extracted in 2 mL of trifluoroacetic acid (TFA, 0.1%) containing 6.3 mM diethylenetriaminepentaacetic acid (DTPA) and 1 mL of 1 mM tris(2-carboxyethyl)phosphate hydrochloride (TCEP) prepared in 200 mM HEPES buffer (pH 8.2) and 6.3 mM DTPA. The homogenate was centrifuged at 12,000 *× g* for 20 min at 4°C. The supernatant was passed through a 0.22-µm membrane filter, and a 250-µL aliquot of the supernatant was mixed with 20 µL of 1 mM TCEP and immediately derivatized with 10 µL of 25 mM monobrombimane, together with 420 µL of 200 mM HEPES buffer containing 6.3 mmol L^–1^ DTPA. Derivatization was conducted in the dark for 30 min at 45°C and terminated by adding 300 µL of 1 M methansulphonic acid (MSA). The thiol derivatives were separated and analyzed by reverse-phase ultra-performance liquid chromatography. The details of the procedure are described elsewhere (Shi et al., 2017).

### Quantitative real-time polymerase chain reaction analysis

Total RNA was extracted from flash-frozen fresh roots using the Promega SV Total RNA Isolation System (Promega) based on the manufacturer’s instructions, and RNA quality was examined by agarose gel electrophoresis and a NanoDrop 2000 spectrophotometer. First-strand cDNA was synthesized from total RNA using M-MLV Reverse Transcriptase (Fermentas). Quantitative reverse transcription-polymerase chain reaction (qRT-PCR) was carried out on a Bio-Rad CFX96 Real-Time PCR system with SYBR Green detection in accordance with the manufacturer’s instructions. The primers used for *TaABCC1* and *TaABCC2* were as reported previously (Bhati et al., 2015), and those for *TaHMA3* were designed according to the sequence KF683296.1 (https://www.ncbi.nlm.nih.gov/). The *tubulin* gene was used as an internal standard control gene (He et al., 2018). The primer sequences are listed in Supplementary **Table S1**. The qRT-PCR reaction conditions comprised pre-denaturation at 95℃ for 1 min followed by 36 cycles of denaturation at 95℃ for 10 s, annealing at 58℃ for 15 s, and extension at 72℃ for 20 s with a final incubation at 72℃ for 5 min. Three technical replications were performed for each sample. The 2^–ΔΔCt^ comparative method was employed to calculate relative gene expression levels (Livak and Schmittgen, 2001).

### Data analysis

Data are expressed as means ± standard deviation of three independent replicates and statistically analyzed by analysis of variance (ANOVA) using SPSS software (v. 25.0). Duncan’s multiple comparison test was used to assess differences among treatments, and a *p*-value *≤* 0.05 was considered indicative of statistical significance. Figures and tables were generated using Excel 2016 and SigmaPlot 12.5 software. The translocation factor (TF), an indicator of the ability of plants to translocate As or Cd from roots to shoots, was calculated as the ratio of As or Cd concentration in shoots to roots (He et al., 2015; Huang et al., 2021).

## Results

### Influence of S on the growth of As- and Cd-treated plants

**Figure 1:**
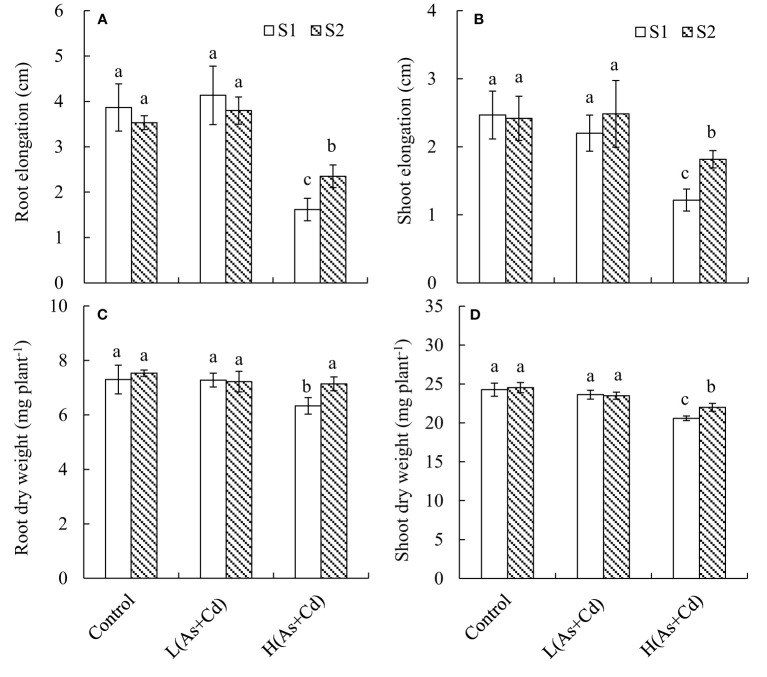
Root **(A)** and shoot **(B)** elongation, and root **(C)** and shoot **(D)** dry weight of wheat plants grown in low S or high S nutrient solution after 6 days of As + Cd exposure. S1, deficient sulfate; S2, sufficient sulfate; L(As+Cd), low As plus Cd; H(As+Cd), high As plus Cd. Data are means ± SD (*n =* 3). Different letters in the same column indicate significant differences among treatments at *p ≤* 0.05 according to Duncan’s multiple comparison test.

We evaluated the impact of S supply and As+Cd treatment on the growth of wheat seedlings in terms of root and shoot elongation and dry weight (**Figure 1**). Compared with the control, the low As+Cd treatment did not significantly alter any of the parameters. However, the high As+Cd treatment caused significant (*p ≤* 0.05) inhibition of plant growth, leading to 58.2%, 44.7%, 13.3%, and 15.2% decreases in root elongation, shoot elongation, root dry weight, and shoot dry weight, respectively, under deficient S supply. By contrast, sufficient S supply significantly alleviated the plant growth inhibition induced by high As+Cd, because the four growth parameters were increased by sufficient compared to deficient S supply. The root elongation, shoot elongation, root dry weight, and shoot dry weight in sufficient S supply were 24.7%, 17.8%, 12.7%, and 6.9% higher than those in deficient S supply, respectively. In the zero (control) and low As+Cd treatments, the differences in the four growth parameters between the two S supplies were not significant.

### Influence of S on As and Cd accumulation and translocation in wheat plants

**Figure 2:**
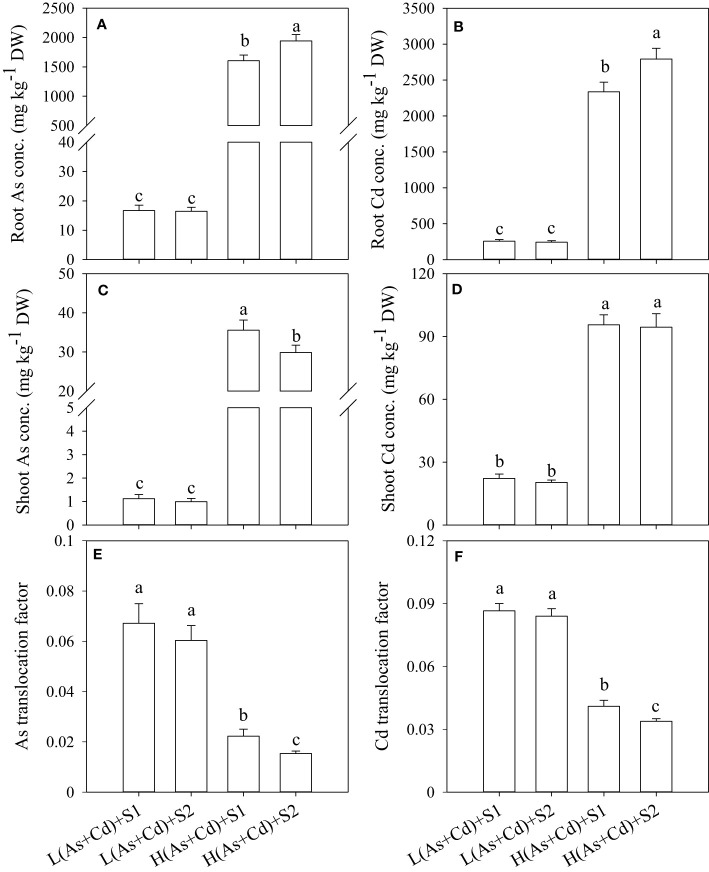
Arsenic and Cd accumulation and translocation in wheat plants grown in low S or high S nutrient solution after 6 days of As plus Cd exposure. S1, deficient sulfate; S2, sufficient sulfate; L(As+Cd), low As plus Cd; H(As+Cd), high As plus Cd. Data are means ± SD (*n =* 3). Different letters in the same column indicate significant differences among treatments at *p ≤* 0.05 according to Duncan’s multiple comparison test.

The concentrations of As and Cd in wheat roots and shoots increased significantly with increasing As and Cd concentrations in nutrient solution (**Figure 2**). Arsenic and Cd accumulated mainly in wheat roots irrespective of S supply. In deficient S- and sufficient S-treated plants, the As and Cd concentrations in wheat roots were 17.9- and 11.7-fold higher than those in shoots in the low As+Cd treatment, and were further increased to 54.8- and 27.0-fold by the high As+Cd treatment, respectively (**Figures 2A–D**). In the low As+Cd treatment, S supply had no significant effect on As and Cd accumulation in wheat plants (**Figures 2A–D**). In the high As+Cd treatment, sufficient S supply increased the root As and Cd concentrations by 13.5% and 19.5%, respectively, in comparison with deficient S supply (**Figures 2A, B**). By contrast, sufficient S supply significantly decreased the shoot As concentration by 15% but had no significant effect on the shoot Cd concentration (**Figures 2C, D**). Sufficient S supply significantly decreased As and Cd translocation from root to shoot in plants treated with high As and Cd concentrations but had no significant effect on As and Cd translocation in wheat plants treated with low As and Cd concentrations (**Figures 2E, F**).

### Influence of S on the subcellular distribution of As and Cd in wheat roots

As shown in **Table 1**, in both low As+Cd- and high As+Cd-treated plants, most As and Cd in root cells was distributed in the cell wall and soluble fractions, and little was in the organelle fraction. In the low As+Cd treatment, S supply had no significant effect on the concentration and proportion of As and Cd in the various subcellular fractions. However, the subcellular distributions of As and Cd varied according to S supply in the high As+Cd treatment. In the high As+Cd treatment, sufficient S supply significantly increased As and Cd concentrations in the soluble fraction by 80% and 108%, respectively, and decreased the As concentration in the organelle fraction by 19%, when compared with deficient S supply. No significant differences were found in the concentrations of As and Cd in the cell wall and the concentration of Cd in the organelle fraction between deficient and sufficient S supply. Therefore, sufficient S supply significantly increased the As and Cd proportions in the soluble fraction compared to deficient S supply. Also, sufficient S supply significantly decreased the proportion of As and Cd in the cell wall and organelle fractions.

**Table 1:** Subcellular distribution of As and Cd in the roots of wheat plants grown in deficient S or sufficient S nutrient solution after 6 days of As plus Cd exposure.

Subcellular fraction	Treatment	Concentration (mg kg^–1^ FW)			Proportion (%)	
		As	Cd		As	Cd
Cell wall	L(As+Cd) +S1	1.06 ± 0.03 b	8.45 ± 0.59 b		58.1 ± 1.5 a	67.8 ± 5.2 a
	L(As+Cd) + S2	0.94 ± 0.19 b	7.89 ± 1.16 b		55.5 ± 3.7 a	66.5 ± 5.4 ab
	H(As+Cd) + S1	63.26 ± 7.60 a	73.20 ± 12.29 a		56.9 ± 7.0 a	74.8 ± 2.3 a
	H(As+Cd) + S2	57.26 ± 9.10 a	81.32 ± 8.13 a		40.7 ± 0.6 b	62.4 ± 0.8 b
						
Soluble fraction	L(As+Cd) +S1	0.73 ± 0.08 c	3.86 ± 0.78 c		39.7 ± 1.9 b	30.8 ± 5.0 ab
	L(As+Cd) + S2	0.69 ± 0.05 c	3.79 ± 1.03 c		41.8 ± 4.3 b	31.6 ± 5.4 ab
	H(As+Cd) + S1	45.05 ± 8.16 b	22.60 ± 1.12 b		40.5 ± 7.2 b	23.3 ± 2.1 b
	H(As+Cd) + S2	81.12 ± 15.27 a	46.93 ± 6.31 a		57.8 ± 0.8 a	36.0 ± 0.8 a
						
Organelle fraction	L(As+Cd) +S1	0.04 ± 0.01 c	0.16 ± 0.03 b		2.1 ± 0.4 ab	1.3 ± 0.2 b
	L(As+Cd) + S2	0.05 ± 0.01 c	0.22 ± 0.07 b		2.7 ± 0.5 a	1.8 ± 0.3 ab
	H(As+Cd) + S1	2.91 ± 0.25 a	1.85 ± 0.07 a		2.6 ± 0.2 a	1.9 ± 0.2 a
	H(As+Cd) + S2	2.37 ± 0.17 b	1.96 ± 0.21 a		1.7 ± 0.2 b	1.5 ± 0.0 b

S1, deficient sulfate; S2, sufficient sulfate; L(As+Cd), low As plus Cd; H(As+Cd), high As plus Cd. Data are means ± SD (n = 3). Different letters in each column indicate significant differences among treatments at p ≤ 0.05 according to Duncan’s multiple comparison test.

### Influence of S on thiol synthesis in wheat roots

**Table 2** shows the concentrations of Cys, GSH, and PCs in roots of wheat plants exposed to various As and Cd concentrations under deficient or sufficient S supply. The concentrations of Cys and GSH in wheat roots were significantly higher in the high As+Cd treatment as compared to the controls, whereas no significant differences were observed between the low and zero As+Cd treatments. High As+Cd exposure induced PC synthesis in wheat roots, and PC_4_ was only detected in high As+Cd-treated plants. The average (of two S treatments) concentrations of PC_2_ and PC_3_ in wheat roots in the high As+Cd treatment were 38.2- and 22.2-fold higher than those in the low As+Cd treatment, and 88.9- and 37.9-fold higher than those in the zero As+Cd treatment, respectively (**Table 2**). Compared to deficient S supply, sufficient S supply had no significant effect on Cys, GSH, and PC concentrations in wheat roots in the zero and low As+Cd treatments, but significantly increased the Cys, GSH, PC_2_, PC_3_, and PC_4_ concentrations in roots by 27%, 51%, 81%, 95%, and 57%, respectively, in the high As+Cd treatment.

**Table 2:** Concentrations of thiol (-SH) compounds in the roots of wheat plants grown in deficient S or sufficient S nutrient solution after 6 days of As plus Cd exposure (nmol SH g^–1^ FW).

Treatment	Cys	GSH	PC_2_	PC_3_	PC_4_
Control (S1)	38.84 ± 3.28 c	3.51 ± 0.07 c	3.11 ± 0.62 d	2.64 ± 0.25 e	ND
Control (S2)	47.57 ± 7.75 c	4.16 ± 0.49 c	2.85 ± 0.57 d	2.84 ± 0.44 de	ND
L(As+Cd) + S1	45.25 ± 6.97 c	3.06 ± 0.29 c	6.53 ± 1.43 c	4.04 ± 0.59 cd	ND
L(As+Cd) + S2	47.73 ± 9.04 c	3.18 ± 0.23 c	7.36 ± 1.42 c	5.33 ± 1.47 c	ND
H(As+Cd) + S1	119.62 ± 3.69 b	19.20 ± 1.21 b	188.83 ± 34.40 b	70.63 ± 14.47 b	11.65 ± 1.26 b
H(As+Cd) + S2	151.95 ± 11.19 a	29.06 ± 5.71 a	340.97 ± 65.19 a	137.45 ± 28.04 a	18.33 ± 3.32 a

S1, deficient sulfate; S2, sufficient sulfate; L(As+Cd), low As plus Cd; H(As+Cd), high As plus Cd; ND, not detected. Data are means ± SD (n = 3). Different letters in the same column indicate significant differences among treatments at p ≤ 0.05 according to Duncan’s multiple comparison test.

### Influence of S on the expression of TaABCC1, TaABBCC2, and TaHMA3 in wheat roots

**Figure 3:**
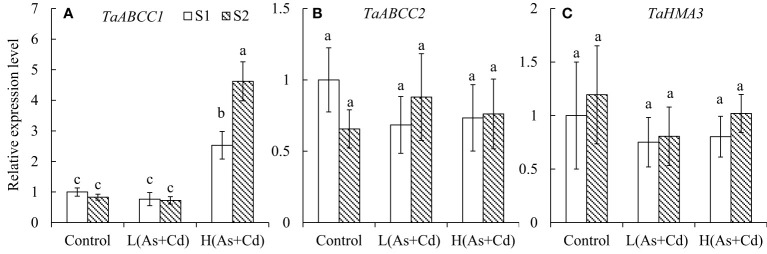
Expression levels of *TaABCC1* **(A)**, *TaABCC2* **(B)**, and *TaHMA3* **(C)** in the roots of wheat plants grown in low S or high S nutrient solution after 6 days of As plus Cd exposure. S1, deficient sulfate; S2, sufficient sulfate; L(As+Cd), low As plus Cd; H(As+Cd), high As plus Cd. Data are means ± SD (*n =* 3). Different letters in the same column indicate significant differences among treatments at *p ≤* 0.05 according to Duncan’s multiple comparison test.

Compared to the control, *TaABCC1* expression in wheat roots was not affected by low As+Cd exposure but was significantly increased by the high As+Cd treatment (**Figure 3A**). The average (of two S treatments) expression level of *TaABCC1* in roots of high As+Cd-treated wheat plants was 4.8-fold higher than that of low As+Cd-treated plants, and 3.9-fold higher than that of zero As+Cd-treated plants (**Figure 3A**). Although sufficient S supply had no significant effect on *TaABCC1* expression in wheat roots in the low and zero As+Cd treatments, it significantly increased *TaABCC1* expression in wheat roots by 83% in the high As+Cd treatment compared with deficient S treatment. *TaABBCC2* and *TaHMA3* expression levels were not significantly affected by S supply or the As+Cd treatments (**Figures 3B, C**).

### Effect of BSO on S-mediated influence on growth and As and Cd translocation in wheat plants

To confirm that S alleviates As and Cd toxicity and root-to-shoot translocation in wheat plants by increasing GSH and PC production under As and Cd stress, BSO was used to manipulate the synthesis of GSH and PCs in high As+Cd-treated plants. As shown in **Table 3**, in the high As+Cd + deficient S treatment, addition of BSO decreased the GSH, PC_2_, PC_3_, and PC_4_ concentrations in wheat roots by 69%, 75%, 84%, and 83%, respectively. In the presence of BSO, the differences in root GSH and PC concentrations between deficient and sufficient S supply were not significant in the high As+Cd treatment. The effects of BSO on plant growth, As and Cd accumulation, the subcellular distribution of As and Cd in roots, and the root-to-shoot translocation of As and Cd in wheat plants exposed to high As+Cd are shown in **Table 4**. Under high As+Cd with deficient S treatment, BSO supply significantly aggravated the toxicity of As and Cd to wheat plants and decreased root elongation, shoot elongation, root dry weight, and shoot dry weight by 59%, 55%, 18%, and 13%, respectively. BSO treatment also decreased As and Cd concentrations in the soluble fraction by 71% and 66%, respectively, and increased the As concentration in the organelle fraction by 31%. BSO treatment decreased the root As concentration by 59%, root Cd concentration by 46%, and shoot Cd concentration by 64%, but had no significant effect on shoot As concentration. Consequently, As and Cd TFs from roots to shoots increased by 2.6- and 1.4-fold, respectively, in response to BSO. Furthermore, in the presence of BSO, the differences in the four plant growth parameters, As and Cd accumulation, the subcellular distribution of As and Cd in roots, and root-to-shoot translocation of As and Cd in wheat plants between deficient S and sufficient S supply were not significant under the high As+Cd treatment.

**Table 3:** Concentrations of thiol (-SH) compounds in the roots of wheat plants grown in deficient S or sufficient S nutrient solution after 6 days of exposure to high As plus Cd with or without BSO (nmol SH g^–1^ FW).

Treatment	Cys	GSH	PC_2_	PC_3_	PC_4_
H(As+Cd) + S1	128.16 ± 14.50 a	19.55 ± 2.04 a	175.70 ± 17.72 a	79.44 ± 12.46 a	10.17 ± 1.80 a
H(As+Cd) + S1 + BSO	111.12 ± 17.54 a	6.11 ± 0.49 b	44.44 ± 4.89 b	12.98 ± 3.45 b	1.72 ± 0.29 b
H(As+Cd) + S2 + BSO	114.39 ± 17.2 a	6.78 ± 0.72 b	47.45 ± 6.50 b	16.74 ± 2.99 b	2.01 ± 0.29 b

S1, deficient sulfate; S2, sufficient sulfate; H(As+Cd), high As plus Cd; BSO, _L_-buthionine sulfoximine. Different letters in each column indicate significant differences among treatments at p ≤ 0.05 according to Duncan’s multiple comparison test.

**Table 4:** Plant growth, As and Cd accumulation, the subcellular distribution of As and Cd in roots, and As and Cd translocation from roots to shoots of wheat plants grown in deficient S or sufficient S nutrient solution after 6 days of exposure to high As plus Cd with or without BSO.

Parameter	H(As+Cd) + S1	H(As+Cd) + S1 + BSO	H(As+Cd) + S2 + BSO
Root elongation (cm)	2.03 ± 0.25 a	0.83 ± 0.15 b	0.93 ± 0.21 b
Shoot elongation (cm)	1.26 ± 0.15 a	0.57 ± 0.06 b	0.63 ± 0.06 b
Root dry weight (mg plant^–1^)	6.56 ± 0.29 a	5.39 ± 0.29 b	5.44 ± 0.35 b
Shoot dry weight (mg plant^–1^)	20.72 ± 0.46 a	18.08 ± 1.26 b	18.97 ± 0.42 b
Root As conc. (mg kg^–1^ DW)	1461.8 ± 176.8 a	601.5 ± 58.7 b	647.7 ± 45.4 b
Shoot As conc. (mg kg^–1^ DW)	32.3 ± 3.4 a	34.7 ± 2.5 a	31.5 ± 3.2 a
Root Cd conc. (mg kg^–1^ DW)	2108.7 ± 241.9 a	974.4 ± 83.1 b	1006.5 ± 102.6 b
Shoot Cd conc. (mg kg^–1^ DW)	91.6 ± 8.2 a	58.2 ± 3.3 b	60.8 ± 5.1 b
As in cell wall (mg kg^–1^ FW)	57.7 ± 6.7 a	34.2 ± 6.3 b	36.6 ± 4.7 b
As in soluble fraction (mg kg^–1^ FW)	39.8 ± 6.8 a	11.6 ± 2.9 b	14.8 ± 1.9 b
As in organelle fraction (mg kg^–1^ FW)	2.99 ± 0.4 b	3.9 ± 0.4 a	3.9 ± 0.3 a
Cd in cell wall (mg kg^–1^ FW)	77.7 ± 6.7 a	47.1 ± 4.6 b	48.4± 4.0 b
Cd in soluble fraction (mg kg^–1^ FW)	29.5 ± 4.1 a	10.0 ± 1.8 b	11.0 ± 1.5 b
Cd in organelle fraction (mg kg^–1^ FW)	1.7 ± 0.2 a	1.8 ± 0.2 a	1.8 ± 0.3 a
As translocation factor	0.022 ± 0.001 b	0.058 ± 0.008 a	0.049 ± 0.004 a
Cd translocation factor	0.044 ± 0.007 b	0.060 ± 0.007 a	0.061 ± 0.007 a

S1, deficient sulfate; S2, sufficient sulfate; H(As+Cd), high As plus Cd; BSO, _L_-buthionine sulfoximine. Data are means ± SD (n = 3). Different letters in the same row indicate significant differences among treatments at p ≤ 0.05 according to Duncan’s multiple comparison test.

## Discussion

It’s well known that the translocation of As and Cd from roots to shoots in plants is species specific and depends on the external metal/metalloid concentration and the intensity of metal/metalloid phytotoxicity (Siemianowski et al., 2011). Therefore, to systematically study the physiological and molecular mechanisms governing the effects of S on As and Cd translocation in wheat, we exposed wheat seedlings to low As+Cd (1 µM As plus 0.5 µM Cd; no significant effect on wheat growth) or high As+Cd (50 µM As plus 30 µM Cd; significant inhibition of plant growth) under deficient S or sufficient S supply. In this study, 6 days of high As+Cd stress resulted in significant reductions in root and shoot elongation and root and shoot dry weight under deficient S supply. Notably, the magnitudes of the reductions in the four growth parameters were less in the high As+Cd treatments with sufficient S supply, suggesting that S enhances the growth of wheat plant under high As+Cd stress. This can be attributed to the alleviating effect of S on As and Cd toxicity in wheat because the growth of plants was not affected by S supply under the zero or low As+Cd treatment (**Figure 1**). These results are consistent with prior reports that S supply improves rice plant growth under As or Cd stress (Srivastava et al., 2016; Cao et al., 2018), but not under control conditions for 14 days (Srivastava et al., 2016). Consistent with other studies on rice (Srivastava et al., 2016) and barley (Reid et al., 2013), this study also showed that 12 days of S deficiency did not have a significant impact on wheat growth under the control conditions. This is not surprising considering the high capacity of plants to buffer changes in shoot and root biomass production in response to S limitation (Elberse et al., 2003). Plants achieve this buffering effect by altering the expression of S metabolism-related hormones and genes (Reid et al., 2013; Srivastava et al., 2016).

The vacuole is the largest plant organelle, and is crucial for detoxification (Martinoia et al., 2012). For normal cell development, plants attempt to sequestrate As and Cd into vacuoles (Song et al., 2014a; Cao et al., 2018). Our results showed that sufficient S supply increased the proportion of As and Cd in the soluble fraction (mainly in vacuoles) of wheat plants grown under the high As+Cd treatment (**Table 1**). This suggests that, under high As+Cd stress, S supply increased vacuolar sequestration of As and Cd. Sequestration of As and Cd into the vacuole decreased their activity in the cytosol (**Table 1**), thereby alleviating As and Cd toxicity (Song et al., 2014a; Cao et al., 2018). This explains why S supply has different effects on plant growth at different As and Cd concentrations (**Figure 1**). Previous study demonstrated that S supply can increase Cd vacuolar sequestration in rice roots by two pathways: 1) transporting Cd^2+^ into vacuoles *via* the OsHMA3 transporter and 2) transporting Cd-PC complexes into vacuoles *via* a PC-dependent pathway (Cao et al., 2018). However, the HMA3 transporter in wheat plants may not transport Cd (Zhang et al., 2020). In this study, *TaHMA3* expression was not affected by the S and As+Cd treatments (**Figures 3B, C**).

PCs play an important role in As and Cd tolerance and translocation in plants (Batista et al., 2014; Zanella et al., 2016; Das et al., 2021). PCs are high-affinity metal/metalloid chelators that can be induced by As and Cd stress in many plants (Shi et al., 2017; Das et al., 2021; González et al., 2021). Similarly, in this study, high As+Cd treatment increased the synthesis of GSH and PCs in wheat roots in comparison with zero and low As+Cd treatment (**Table 2**). Supply of S further increased the synthesis of GSH and PCs under high As+Cd stress (**Table 2**), possibly enhancing As and Cd tolerance and reducing As and Cd translocation in wheat plants (**Figures 1**, **2**). By contrast, S supply in the zero and low As+Cd treatments had no significant impact on the Cys, GSH, and PC concentrations in wheat roots (**Table 2**), and thus likely had no significant effect on As and Cd accumulation and translocation in wheat (**Figure 2**). In addition, the suppression of GSH and PC synthesis by BSO hypersensitized wheat plants to As and Cd toxicity, and increased root-to-shoot As and Cd translocation (**Tables 3**, **4**). The differences in plant growth, the subcellular distribution of As and Cd in roots, and the root-to-shoot translocation of As and Cd between deficient S and sufficient S supply were not significant in the presence of BSO (**Table 4**), although they differed significantly in its absence (**Figures 1**, **2**). Taken together, these results indicate that S decreased As and Cd translocation and their toxicity to wheat plants mainly by increasing the synthesis of GSH and PCs, which then chelated more As and Cd and promoted As and Cd transfer into vacuoles (**Table 1**).

After chelating with PCs, the complexes of As-PCs and Cd-PCs should be sequestered into plant vacuoles for final detoxification (Song et al., 2010; Park et al., 2012). However, due to the large size and polyploid complexity of the wheat genome, the transporters involved in the vacuolar sequestration of As-PCs and Cd-PC in wheat plants are unknown. In Arabidopsis, two transporters (AtABCC1 and AtABCC2) of the ATP-binding cassette transporter family transport As-PCs and Cd-PCs into the vacuole (Song et al., 2010; Park et al., 2012). Similarly, OsABCC1 is the main transporter for the vacuolar sequestration of As-PCs complexes in rice (Song et al., 2014b). In wheat, only one gene, *TaABCC1*, shows high similarity to *AtABCC1* and *AtABCC2* (Bhati et al., 2015). In this study, *TaABCC1* expression in wheat roots was significantly induced by the high As+Cd treatment, which was further enhanced by sufficient S supply. Furthermore, the changes in *TaABCC1* expression under various As+Cd and S conditions were consistent with the changes in GSH and PCconcentrations in wheat roots, which were correlated with As and Cd translocation and plant tolerance to As and Cd. Therefore, S supply increases As and Cd vacuolar sequestration in wheat roots by increasing PC synthesis and *TaABCC1* expression, suggesting a mechanism by which S regulates As and Cd translocation and toxicity in wheat.

## Conclusion

Sufficient S supply simultaneously decreased As and Cd translocation and their toxicity in wheat plants under As and Cd stress. This could be attributed to induction of the synthesis of PCs and the expression of *TaABCC1*, increasing As and Cd chelation and transfer into vacuoles. Furthermore, the HMA3 transporter in wheat roots is not involved in S-mediated regulation of Cd vacuolar sequestration, even through this transporter is crucial for S-induced Cd vacuolar sequestration in rice (Cao et al., 2018). This study sheds light on the physiological and molecular mechanisms by which S modulates As and Cd translocation in wheat and will facilitate the development of strategies to simultaneously decrease the accumulation of As and Cd in wheat grain.

## Data availability statement

The original contributions presented in the study are included in the article/**Supplementary Material**. Further inquiries can be directed to the corresponding authors.

## Author contributions

GS, LL, and YG conceived and designed this study. HL, HZ, GF, and WC conducted the experiment. HL, HZ, and JL analyzed the data and prepared the tables and figures. GS and LL wrote the first draft of the manuscript. DZ and YG reviewed and edited the manuscript. All authors contributed to the article and approved the submitted version.

## Funding

This work was funded by the Key Research and Development Plan of Jiangsu Province (BE2021717), the Jiangsu Agricultural Science and Technology Independent Innovation Fund (CX(20)1010), and the National Natural Science Foundation of China (41601541).

## Acknowledgments

The authors are grateful to Ms. Xin Wang for her technical assistance with ICP-MS analysis.

## Conflict of interest

The authors declare that the research was conducted in the absence of any commercial or financial relationships that could be construed as a potential conflict of interest.

## Publisher’s note

All claims expressed in this article are solely those of the authors and do not necessarily represent those of their affiliated organizations, or those of the publisher, the editors and the reviewers. Any product that may be evaluated in this article, or claim that may be made by its manufacturer, is not guaranteed or endorsed by the publisher.
