# Supplementary material for: Sulfur reduces the root-to-shoot translocation of arsenic and cadmium by regulating their vacuolar sequestration in wheat (Triticum aestivum L.)
Source: Front Plant Sci. 2022 Oct 5;13:1032681. doi: 10.3389/fpls.2022.1032681 (PMC9580998; doi:10.3389/fpls.2022.1032681)
Supplement: Supplementary file 1 [file DataSheet_1.doc]

Supplementary Material

| **Supplementary Table S1**. Primers used in this study. | | |
| --- | --- | --- |
| Gene name | Primer sequences | |
|  | Forward (5'-3') | Reverse (5'-3') |
| TaABCC1 | GTCTGTCCACAATCCGTGCTT | TCATGATGCCACCCAATGTT |
| TaABCC2 | GGGAGTGCATGATTTGCGGTGTAGG | TACCCTGGCCAAGCACACAAGTTGCC |
| TaHMA3 | TCTTCATCCGCCCGCGTCTC | CCGTTGAGGACCTTCACAAT |
| TaTubulin | GTGGAACTGGCTCTGGC | CGCTCAATGTCAAGGGA |

**Supplementary** **Fig. S1**. Relative root elongation (RRE) of wheat plants grown in deficient S nutrient solution after exposure to the indicated As or Cd concentrations for 6 days. Relative root elongation is the root length ratio of treated and non-treated wheat. Data are means ± SD (*n =* 3). Bars with different letters indicate significant differences at *p ≤* 0.05 according to Duncan’s multiple comparison test.
